# Human Acquired Aplastic Anemia Patients' Bone-Marrow-Derived Mesenchymal Stem Cells Are Not Influenced by Hematopoietic Compartment and Maintain Stemness and Immune Properties

**DOI:** 10.1155/2021/6678067

**Published:** 2021-04-29

**Authors:** Vandana Sharma, Sonali Rawat, Suchi Gupta, Sweta Tamta, Rinkey Sharma, Tulika Seth, Sujata Mohanty

**Affiliations:** ^1^Department of Hematology, All India Institute of Medical Sciences, New Delhi 110029, India; ^2^Stem Cell Facility, DBT-Centre of Excellence for Stem Cell Research, All India Institute of Medical Sciences, New Delhi 110029, India

## Abstract

**Methods:**

In the current study, we investigated the morphological differences, proliferation capacity, population doubling time (PDT), surface marker profiling, trilineage differentiation potential, and immunosuppressive ability of BM Mesenchymal Stem Cells (BM-MSCs) from untreated aAA patients and in the same number of age- and gender-matched controls.

**Results:**

We observed similar morphology, proliferation capacity, phenotype, trilineage differentiation potential, and immunomodulatory properties of BM-MSCs in aAA patients and control subjects.

**Conclusion:**

Our results confirm that the basic and immunosuppressive properties of BM-MSCs from aAA patients do not differ from normal BM-MSCs. Our data suggest that BM-MSCs from aAA patients might not be involved in disease pathogenesis. However, owing to a smaller number of samples, it is not conclusive, and future studies with more exhaustive investigation at transcriptome level are warranted.

## 1. Introduction

The characteristic features of acquired aplastic anemia (aAA) are peripheral blood (PB) pancytopenia and bone marrow (BM) hypocellularity. It is believed that autoreactive T cells destroy hematopoiesis in aAA [1]. Apart from this, there are qualitative and quantitative defects of stem cells in BM of aAA patients [2]. BM stroma consists of different cell populations of hematopoietic and nonhematopoietic stem cells [3]. The nonhematopoietic progenitor cells are called bone marrow mesenchymal stem cells (BM-MSCs) [4]. MSCs are self-renewing and can differentiate into multilineages such as adipocytes, chondrocytes, and osteocytes [5]. MSCs affect hematopoietic stem cells and immune cells including T cells by cytokine secretion and direct cell-to-cell interaction [6–8].

The underlying pathogenetic mechanism of acquired aplastic anemia involves inefficient hematopoiesis and abnormal immune responses. Hence, alterations in bone-marrow-derived mesenchymal stem cells could primarily or secondarily lead to acquired aplastic anemia. Features of altered BM microenvironment have been described in acquired aplastic anemia [5, 9]. Previous studies on BM-MSCs in aAA have shown equivocal outcomes, such as abnormal morphology [10, 11], lower population doubling time, and poor proliferation and differentiation capacity of aAA BM-MSCs [10, 12–17], whereas others have found no differences in aAA BM-MSCs as compared to normal BM-MSCs [18–20]. Thus, the exact picture of BM microenvironment and its role in the disease need investigation.

To understand the difference between the characteristics of acquired aplastic anemia BM-MSCs and normal healthy BM-MSCs, the following study was designed. In this study, we have evaluated the morphology, proliferation capacity, population doubling time, surface marker profiling, and differentiation potential of BM-MSCs from aAA patients compared to normal BM-MSCs. We have also evaluated the immunomodulatory potential of these MSCs as it is one of the important mechanisms by which MSCs show their repairable and regenerative potential.

## 2. Materials and Methods

Five untreated aAA patients diagnosed as per the standard international criteria (Camitta et al.) [21] were enrolled from the Department of Hematology, All India Institute of Medical Sciences (AIIMS), New Delhi, India. The patients were stratified into nonsevere, severe, and very severe aAA. Signed informed consent was taken from all the study subjects. 1 ml of bone marrow sample was collected from all the patients undergoing the routine medical test procedure. This study was approved by the Institutional Ethics Committee of AIIMS, New Delhi, India (Ref: IEC/T-353/30/08/13).

### 2.1. Isolation and Expansion of Bone Marrow Mesenchymal Stem Cells

MSCs were isolated and cultured as described previously [22, 23]. Unmanipulated bone marrow was seeded in 60 mm culture dish (BD, USA) in complete growth media containing 1X Dulbecco's Modified Eagle Medium-Low Glucose (DMEM-LG) (Life Technologies, USA) media with 10% Fetal Bovine Serum (FBS) (HyClone, USA), 1% Penicillin (100U/ml) + Streptomycin (100 *μ*g/ml) (Life Technologies, USA). The cells were incubated in a humidified atmosphere at 37°C with 5% CO_2_. Medium was changed every 3 days until the cell confluency reached 80%. Adherent cells were then passaged with 0.25% trypsin-EDTA (Invitrogen, Gibco) and reseeded at 1 × 10^4^ cell/cm^2^.

### 2.2. Trilineage Differentiation

Mesenchymal Stem Cells are multipotent cells which can be differentiated into osteocytes, adipocytes, and chondrocytes. Therefore, the healthy and aAA MSCs were characterized for their trilineage differentiation potential as per the previous established protocols of our laboratory [24].

### 2.3. Population Doubling Time (PDT)

MSCs for each sample (*N* = 3) were seeded at a density of 50 × 10^3^ cells per 35 mm petri dish (Becton Dickinson, USA). After 72 hrs, MSCs were enumerated and assessed for viability using Trypan Blue dye exclusion (Life Technologies, USA) assay. The PDT was obtained by the following formula [24]:

(1) $$\begin{array}{c} \text{PDT}=T-T_{\text{o}}\text{ Log }2\left(\text{Log }N-\text{Log}N_{o}\right), \end{array}$$

where *T* is the time of harvesting, *T*_o_ is the time of seeding, *N* is the number of cells harvested, and *N*_o_ is the number of cells seeded.

### 2.4. Measurement of Metabolic Activity by MTT Assay

Proliferation rate of hMSCs (*N* = 3) was performed at days 1, 3, 5, 7, and 14 and measured by 3-(4,5-dimethylthiazol-2-yl)-2,5-diphenyltetrazolium bromide (MTT) (Sigma, USA) assay. The technique was performed as per the previous established protocol [12].

### 2.5. Immunophenotyping

At passage 3, cells were characterized using monoclonal antibodies specific for CD105-APC, CD73-PE, CD29-FITC, CD90-PerCp-Cy5.5, HLA-ABC-APC, HLA-DR-FITC, and CD34/45-PE/FITC (BD Pharmingen, France). Acquisition and data analysis were performed using flow cytometry BD-LSR-II (BD Biosciences, France) and FACS Diva Software Version 6.2.

### 2.6. Immunofluorescence

Immunofluorescence studies were performed for the detection of intracellular Vimentin protein in the MSC samples. Trypsinised cells were seeded on coverslips kept in a 35 mm dish (BD Biosciences, USA), and MSC expansion media were added and incubated at 37°C, 5% CO_2._ Once confluency reached within 40–50%, the existing media from the dish were removed and cells were washed with PBS. Cells were fixed with 1 ml of 4% paraformaldehyde for 20 mins at 4°C. Permeabilization and blocking was done with 0.05% Tween-20 followed by 2% BSA in PBS for 30 mins at room temperature. MSCs were incubated with the Mouse monoclonal vimentin (Abcam, Cambridge, USA) primary antibody (1/100) for overnight at 4°C. Next day, after washing with PBS, cells were incubated with the secondary fluorescent antibody (1/500) for 40 mins at room temperature (RT). To visualise nuclei, slides were stained with diluted 1/4000 DAPI (Life Technologies, USA) for 3 mins at RT followed by thorough washing of the cells with PBS. The acquisition and imaging of the cells were performed using Floid Microscopy (Life Technologies).

### 2.7. Immunosuppressive Ability of MSCs: One-Way Mixed Lymphocyte Reaction

The study was approved by the Institutional Ethics Committee (IEC) (Ref no. IECPG-345/07.09.2017 (RT-6/29.11.2017)). All the samples were obtained after taking the donor's informed consent. Human Peripheral Blood Mononuclear Cells (PBMCs) were isolated by Ficoll-Paque (Axis-Shield; Oslo, Norway) density gradient centrifugation from blood donated by healthy volunteers. Phytohemagglutinin A (PHA (Sigma, USA); 35 *μ*g/mL) was used to stimulate the activation of human peripheral blood mononuclear cells (PBMCs) before coculture [25]. For coculture experiments, hMSCs were treated with Mitomycin C (Sigma, USA); 15 *μ*g/ml and cocultured (1 × 10^4^ cells/well) with PHA activated hPBMCs (5 × 10^4^ cells/well) in 1 : 5 ratio in RPMI-1640 medium (Gibco, USA) containing 10% FBS for 3 days in 96-well plates (Costar, USA). Proliferation of hPBMCs was assessed by MTS assay. The 200 *μ*l cell culture supernatant containing hPBMCs was collected in prelabelled 0.6 ml Eppendorf tube, and 20 *μ*l of MTS reagent (Promega, USA) was added in each tube, followed by incubation for 3 h at 37°C and 5% CO_2_. Afterwards, the tubes were centrifuged at 300*g* for 5 mins. 200 *μ*l of supernatant was collected from each tube and transferred into the fresh 96-well plate. The absorbance was taken at 490 nm using ELISA reader (BioTek, Germany). Lastly, % decrease was calculated by calculating the difference between positive control (activated PBMCs) and test group (MLR). Then, we divided the decrease by the positive control and multiplied the answer by 100.

## 3. Statistical Analysis

Data analysis was performed using Prism 5 (GraphPad) and Excel (Microsoft). A statistically significant difference among groups was determined by *t*-test or one-way analysis of variance (ANOVA). The cutoff value of significance (*P* value) was 0.05. Results were expressed as mean ± SD.

## 4. Results

### 4.1. Morphological Analysis and Immunophenotypic Profile of aAA-MSCs and Control-MSCs

A total of 10 subjects, five aAA donors and five controls, were enrolled in the study. Clinical data of these donors are outlined in Table 1. The average age was 20.4 years. All BM aspirates were obtained at the time of diagnosis before therapy was started.

**Figure 1:**
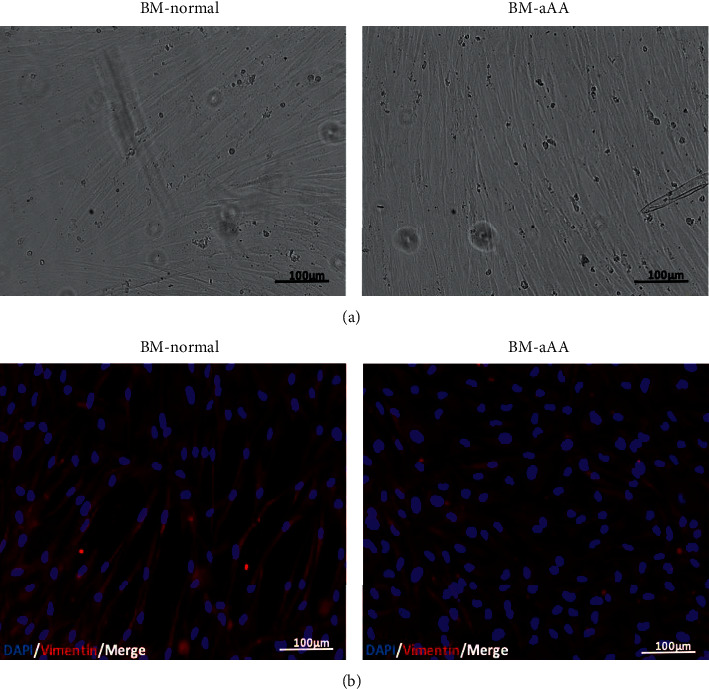
Representative images for BM-MSCs. (a) A micrograph to show typical spindle-shaped morphology of BM-MSCs from normal donor versus aplastic anemia. (b) An immunofluorescence image for vimentin expression level of vimentin in BM-normal vs. BM-aAA.

**Figure 2:**
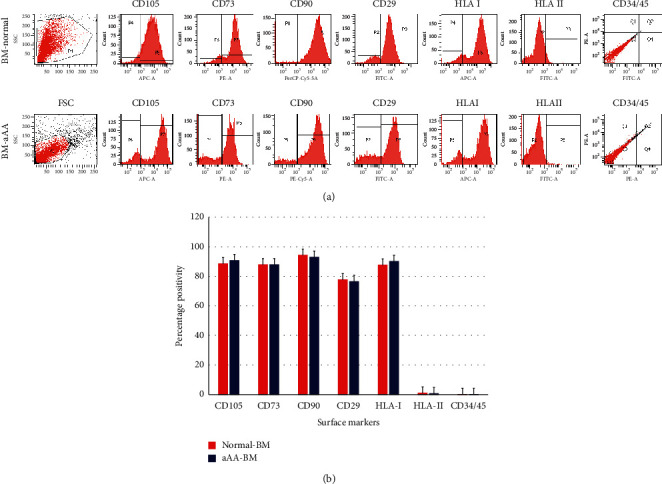
Immunophenotypic analysis of mesenchymal stem cells from BM-aAA and BM-normal. (a) Representative histograms for a panel of surface marker profiling. (b) A cumulative graph comparing the surface marker expression in normal MSCs and MSCs from aplastic anemia.

MSCs of aAA and control group shared a similar spindle-shaped morphology *in vitro* (Figures 1(a) and 1(b)). Both revealed a consistent immunophenotypic profile which was negative for CD34/CD45. HLA-DR was positive for CD105, CD90, CD29, CD73, and HLA-ABC (Figures 2(a) and 2(b)). No significant difference was noted in the expression of any single surface marker between the two study groups.

### 4.2. Growth Kinetics of aAA-BM-MSCs and Normal-BM-MSCs

**Figure 3:**
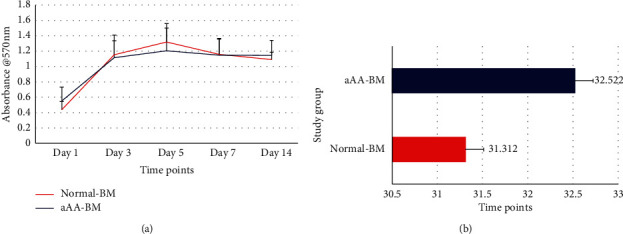
The representative data show the comparison of the proliferative index of aAA-BM and Normal BM. (a) A line graph representing the average metabolic activity of normal BM and aAA-BM (*n* = 5 for each group) at different time points. (b) A bar graph representing the comparison of cumulative PD time at passage 3, shown as mean and 95% confidence interval. *N* = 5.

To determine the growth kinetics of aAA-MSCs and normal MSCs, proliferation was assessed from day 1 to day 14 by MTT assay (Figure 3(a)). We used cells from passages 3 to 5 in order to avoid hematopoietic stem cell contamination. This also prevents adulteration of senescent or differentiating MSCs in later passages [19]. aAA group showed similar expansion rate of proliferation as compared to control group. To further confirm the growth kinetics, another set of cells was passaged to assess the population doubling time (PDT), which was 31 ± 1.5 hrs for aAA MSC and 30 ± 2.10 hrs for control group MSCs (Figure 3(b)).

### 4.3. Differentiation Potential of aAA-MSCs and Normal-MSCs

**Figure 4:**
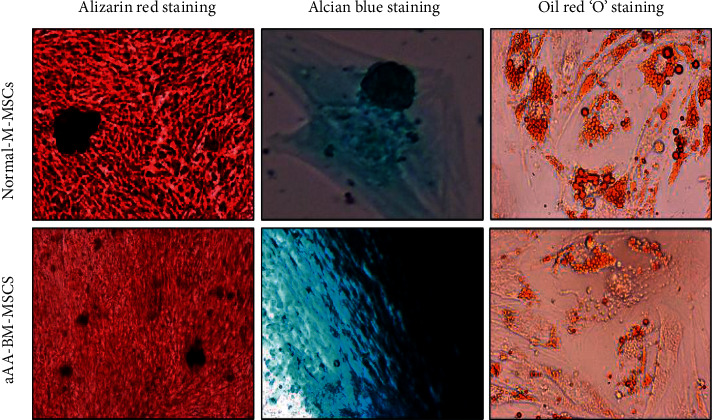
Representative images for trilineage differentiation of BM-MSCs from normal and aAA patients. Alizarin red staining represents the mineralization and signifies the osteogenic differentiation. Alcian blue staining represents the chondrocytes differentiation whereas oil red “O” staining represents the differentiation to adipocytes. Upper panel: normal BM-MSCs. Lower panel: aAA BM-MSCs.

MSCs from patients and normal donors were exposed to differentiation media (Figure 4). In osteogenic conditions, aAA-MSCs could not differentiate into osteocytes as robustly as control MSCs. This was evident by lower mineralization and less intense alizarin red staining. Under adipogenic environment, aAA-MSCs had fewer lipid containing cells, while in control-MSCs, there were larger fat droplets in a single adipocyte. Oil red “O” staining was used for the identification of neutral fat vacuoles. However, the overall osteogenic and adipogenic characteristics of aAA-MSCs and control MSCs did not show a significant difference. In chondrogenic differentiation media, aAA-MSCs and control MSCs showed similar ability to differentiate into chondrocytes. However, the characteristics of normal MSCs and aAA-MSCs did not show significant differences.

### 4.4. Immunosuppressive Ability of aAA-BM and Normal BM-MSCs

**Figure 5:**
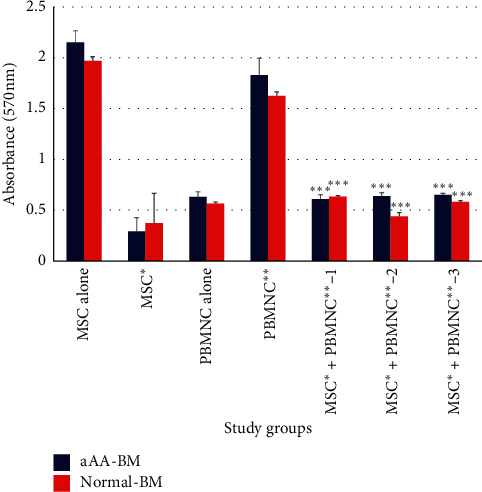
Bar graph representation of one-way mixed lymphocytes reaction (MLR) after establishing cocultures of PBMCs, normal BM, PBMCs, and aAA-BM (*n* = 3). PBMNC^*∗∗*^ corresponds to PHA stimulated PBMNCs versus MSC^*∗*^ + PBMNC^*∗∗*^ (coculture) from all three donors. *P* value <0.005 versus PBMNC^*∗∗*^ with MSCs^*∗*^ + PBMNC^*∗∗*^1,2,3.

The observed results of our experiments suggested that aAA-BM and normal BM-MSCs were able to suppress the proliferation of PBMNCs in coculture experiments. At an MSC:PBMNC ratio of 1:5, the inhibition of proliferation caused by the MSCs was quite significant. However, the calculated percentage of immune suppression by aAA-MSCs and normal MSCs showed that it was similar in both sources of BM-MSCs (Figure 5). The current study states comparable results of MLR experiments, with the percentage decrease of immune cell proliferation being 66.70%, 65.06% and 64.46% for aAA-BM-MSCs in each individual patient, whereas normal BM-MSCs showed 61.13%, 72.93%, and 64.20% of suppression (Table 2).The differences in the immunomodulatory properties of the three samples of aAA-BM and normal-BM have been suggested to be primarily due to quantitative aspects of the suppression, which are likely to be related to the respective metabolic activities of the tissue source. Our data indicate that aAA-BM are similar to normal BM-MSCs in their functional immunological property and retain their ability to suppress allogenic immune cells.

## 5. Discussion

Mesenchymal Stem Cells (MSCs) are multipotent cells that may be isolated from the bone marrow (BM), adipose tissue, dental pulp, umbilical cord blood, or umbilical cord and they are known to contribute to the organization and functioning of the hematopoietic niche. Endothelial cells are known source of MSCs in BM niche and regulate hematopoietic stem cell (HSCs) proliferation and differentiation. However, according to the International Society for cellular therapy, the cells should be able to adhere to plastic surface, express surface markers (positive for CD105, CD90, CD73, and CD29 and negative for CD34, CD14, CD45, and HLA-DR) [26]. MSCs are capable of differentiating into fibroblast, osteoblasts, adipocytes, and chondroblasts. Hence, in the present study, MSCs from aAA patients and healthy donors were compared for cellular morphology, surface marker profiling, population doubling time, and trilineage differentiation potential.

We found spindle-shaped typical morphology of MSCs in aAA and healthy donors. Additionally, our aAA and healthy BM-MSCs expressed vimentin protein. Vimentin protein plays a vital role in cellular stability and is generally expressed in normal mesenchymal cells [25]. Similarly, many studies have reported similar spindle-shaped and fibroblastic morphology of aAA-MSC [13, 19, 27–29]. On the contrary, in some studies, aAA-MSCs were irregularly shaped and swollen [10, 30].Morphology of aAA-MSCs also varied from healthy controls in cultures infected with short hairpin containing lentiviruses [11]. The morphological variation indicates that there might be other responsible factors such as *in vivo* defect or different *in vitro* conditions.

aAA and healthy control MSCs were then characterized using cell surface marker profiling. MSCs were identified based on the minimal criteria of the International Society of Cellular Therapy (ISCT) for human MSCs [26]. The MSCs were negative for CD34/CD45 and HLA-DR and positive for CD105, CD90, CD29, CD73, and HLA-ABC.

Michelozzi and colleagues reported no difference in population doubling time of aAA-BM and normal-BM [29], whereas others have shown lower population doubling time and decreased proliferation in aAA BM-MSCs [13, 19, 27]. In a study, aberrantly expressed genes associated with cellular proliferation, differentiation, and apoptosis from aAA- BM-MSCs were implicated in lower proliferation [30]. Low level of fibroblastic growth factor (FGF2) was also shown to affect functions and growth of aAA-MSCs [28]. However, when we compared the aAA-BM and normal-BM, we did not find any ambiguities in proliferation and population doubling time. It has been observed that the qualities of the growth surface and expansion media can greatly affect cell behavior [14]. This could be the reason for variable growth capacities of MSCs in different studies.

Trilineage differentiation is a hallmark characteristic for identifying healthy and functionally active MSCs. Trilineage differentiation is comprised of osteocytes, adipocytes, and chondrocytes differentiation. Li et al. have reported easier adipogenic differentiation, while it was difficult to induce osteogenic differentiation in aAA-MSCs. Likewise, superior adipocyte differentiation was detected in aAA-MSCs, but osteocyte differentiation was same as controls or was repressed [15, 30]. Other studies have found impaired or decreased adipocytes and osteogenic differentiation in aAA- MSCs than controls [11, 16, 19, 27]. Therefore, it is possible that interaction between adipogenesis and osteogenesis might influence hematopoiesis. Thus, the stress induced by the imbalance of adipocytes and osteocytes might also impact the hematopoietic stem cells in these cases [1], whereas some studies did not find any difference between the differentiation potential of aAA and control MSCs [17, 20, 28, 29]. In accordance with the above reported studies, we observed that aAA patient and healthy MSCs effectively differentiated into osteocytes, adipocytes, and chondrocytes. Although the differentiation into osteocytes and adipocytes in aAA patients was lesser than healthy controls, it was not significant.

The disparities in various studies can be attributed to the variations in patient population and the heterogeneity of acquired aplastic anemia. Overall, a plethora of studies have reported contradicting observations, where aAA-MSCs have been shown to behave like MSCs from healthy donors while others have shown functionally impaired MSCs [13, 15–17, 19, 20, 27–31].

In the present study, at basic parameters, these MSCs show similar trend in terms of cell morphology, proliferation, population doubling time, and trilineage differentiation potential. Apart from these, MSCs are unique in terms of their immune response during inflammation via immunomodulatory factors and release of growth factors, chemokine and anti-inflammatory cytokines [32].

It is already established that MSCs help in tissue repair and prevention of graft-versus-host disease [33]. Altogether, studying and comparing immunomodulatory property of aAA-MSCs and normal MSCs is an essential aspect to understand the functionality of the aAA-MSCs. Xu et al. and Bueno et al. have shown comparable functional immunomodulatory properties of aAA-MSCs. Shipounova et al. observed the differences in the immunomodulatory properties of aAA-BM and normal-BM. Recently, Huo et al. have also found abnormal immunoregulation of aAA MSCs. This could be primarily due to quantitative aspects of the suppression, which are likely to be related to the respective metabolic activities of the tissue source.

In the present study, the immunosuppressive potential of both the aAA and normal MSCs was maintained. Although minute differences were observed, the immunosuppressive ability of MSCs from aAA patients was not equivalent to those obtained from healthy donors.

BM-MSCs have been used in multiple studies and are also being evaluated at clinical level. However, obtaining bone marrow samples from healthy donor is not an easy task. Bone marrow aspiration is a painful process. There are a lot of ethical concerns with respect to bone marrow aspiration from healthy donors for MSCs isolation and expansion. In a routine scenario, bone marrow is obtained from patients undergoing aspiration as a part of routine treatment. During this aspiration, a small amount of bone marrow is required for MSCs isolation. However, there are certain queries related to the MSCs isolated from these patients.

In aAA condition, their will be a defective hematopoiesis which eventually leads to empty bone marrow [34]. The process of hematopoiesis is closely connected with mesenchymal stromal microenvironment of the bone marrow tissue [35]. Consequently, any flaws in bone marrow mesenchymal stromal cells can affect hematopoiesis. There is an urgent need to study MSCs from both donors (healthy versus aAA patient) in great depth both at transcriptional and translational level.

In conclusion, we have shown that BM-MSCs obtained from aAA patients and controls share almost the same morphological, phenotypical, growth, and differential properties; However, they differ in their immunosuppressive properties. Our results suggest that mesenchymal stem cells might not be involved in the pathogenesis of acquired aplastic anemia. Despite being not conclusive owing to the small number of samples, this study can be used as a base for further investigations. With future in-depth analysis and preclinical studies, much more therapeutic potential of MSCs can be explored. The multifaceted pathophysiology of acquired aplastic anemia is responsible for differences in results across various studies. The crosstalk between various components of bone marrow and disease pathogenesis is a fascinating area of research. Therefore, further investigations on stromal components in bone marrow of acquired aplastic anemia patients would be necessary for better understanding of the mechanism of this disease.

## Figures and Tables

**Table 1:** Characteristics of acquired aplastic anemia patients.

S. no.	Age (in years)	Sex	Haemoglobin (g/l)	Total leukocyte count (10^9^/l)	Platelet count (10^9^/l)	Absolute neutrophil count (10^9^/l)	Disease severity
1	24	M	54	2.5	59	0.5	Nonsevere AA
2	19	M	53	2.3	19	0.14	Very severe AA
3	19	F	100	2.22	38	1	Nonsevere AA
4	19	F	50	4.5	55	0.54	Nonsevere AA
5	21	M	54	3.37	13	0.2	Very severe AA

**Table 2:** Tabular representation of the comparative one-way mixed lymphocyte reaction data for the three MSC donors from normal and aAA patients, displaying the result as % decrease of immune cells *P* value <0.005.

Study groups	OD	% decrease-aAA-BM	OD	% decrease- Normal-BM
PBMNC^*∗∗*^	1.829	—	1.626	—
MSC^*∗*^+PBMNC^*∗∗*^-1	0.608	66.70	0.632	61.13
MSC^*∗*^+PBMNC^*∗∗*^-2	0.639	65.06	0.44	72.93
MSC^*∗*^+PBMNC^*∗∗*^-3	0.65	64.46	0.582	64.20

## Data Availability

All data generated and analysed during this study are included within the article. The data are also available from the corresponding author upon request.

## References

[1] Nakao S., Feng X., Sugimori C. (2005). Immune pathophysiology of aplastic anemia. *International Journal of Hematology*.

[2] Rizzo S., Scopes J., Elebute M. O., Papadaki H. A., Gordon-Smith E. C., Gibson F. M. (2002). Stem cell defect in aplastic anemia: reduced long term culture-initiating cells (LTC-IC) in CD^34+^ cells isolated from aplastic anemia patient bone marrow. *The Hematology Journal*.

[3] Lucas D. (2017). The bone marrow microenvironment for hematopoietic stem cells. *Advances in Experimental Medicine and Biology*.

[4] Smith J. N. P., Calvi L. M. (2013). Concise review: current concepts in bone marrow microenvironmental regulation of hematopoietic stem and progenitor cells. *Stem Cells*.

[5] Schrezenmeier H., Jenal M., Herrmann F., Heimpel H., Raghavachar A. (1996). Quantitative analysis of cobblestone area-forming cells in bone marrow of patients with aplastic anemia by limiting dilution assay. *Blood*.

[6] Di Nicola M., Carlo-Stella C., Magni M. (2002). Human bone marrow stromal cells suppress T-lymphocyte proliferation induced by cellular or nonspecific mitogenic stimuli. *Blood*.

[7] Wagner W., Roderburg C., Wein F. (2007). Molecular and secretory profiles of human mesenchymal stromal cells and their abilities to maintain primitive hematopoietic progenitors. *Stem Cells*.

[8] Cuerquis J., Romieu-Mourez R., François M. (2014). Human mesenchymal stromal cells transiently increase cytokine production by activated T cells before suppressing T-cell proliferation: effect of interferon-*γ* and tumor necrosis factor-*α* stimulation. *Cytotherapy*.

[9] Scopes J., Bagnara M., Gordon-Smith E. C., Ball S. E., Gibson F. M. (1994). Haemopoietic progenitor cells are reduced in aplastic anaemia. *British Journal of Haematology*.

[10] Huo J., Zhang L., Ren X. (2020). Multifaceted characterization of the signatures and efficacy of mesenchymal stem/stromal cells in acquired aplastic anemia. *Stem Cell Research & Therapy*.

[11] Chao Y.-H., Wu K.-H., Chiou S.-H. (2015). Downregulated CXCL12 expression in mesenchymal stem cells associated with severe aplastic anemia in children. *Annals of Hematology*.

[12] Kakkar A., Sharma P., Sankar M. M., Kharbanda O., Mohanty S. (2016). Effect of hypoxia on stemness and differentiation of dental pulp derived stem cells. *IOSR Journal of Dental and Medical Sciences*.

[13] Hamzic E., Whiting K., Gordon Smith E., Pettengell R. (2015). Characterization of bone marrow mesenchymal stromal cells in aplastic anaemia. *British Journal of Haematology*.

[14] Salzig D., Leber J., Merkewitz K., Lange M. C., Köster N., Czermak P. (2016). Attachment, growth, and detachment of human mesenchymal stem cells in a chemically defined medium. *Stem Cells International*.

[15] Li J., Lu S., Yang S. (2012). Impaired immunomodulatory ability of bone marrow mesenchymal stem cells on CD^4+^ T cells in aplastic anemia. *Results in Immunology*.

[16] Shipounova I. N., Petrova T. V., Svinareva D. A., Momotuk K. S., Mikhailova E. A., Drize N. I. (2009). Alterations in hematopoietic microenvironment in patients with aplastic anemia. *Clinical and Translational Science*.

[17] Xu Y., Takahashi Y., Yoshimi A., Tanaka M., Yagasaki H., Kojima S. (2009). Immunosuppressive activity of mesenchymal stem cells is not decreased in children with aplastic anemia. *International Journal of Hematology*.

[18] Rawat S., Srivastava P., Prabha P., Gupta S., Kanga U., Mohanty S. (2018). A comparative study on immunomodulatory potential of tissue specific hMSCs: role of HLA-G. *IOSR Journal of Dental and Medical Sciences-ISSN*.

[19] Chao Y.-H., Peng C.-T., Harn H.-J., Chan C.-K., Wu K.-H. (2010). Poor potential of proliferation and differentiation in bone marrow mesenchymal stem cells derived from children with severe aplastic anemia. *Annals of Hematology*.

[20] Bueno C., Roldan M., Anguita E. (2014). Bone marrow mesenchymal stem cells from patients with aplastic anemia maintain functional and immune properties and do not contribute to the pathogenesis of the disease. *Haematologica*.

[21] Camitta B. M., Storb R., Thomas E. D. (1982). Aplastic anemia. *New England Journal of Medicine*.

[22] Nandy S. B., Mohanty S., Singh M., Behari M., Airan B. (2014). Fibroblast growth factor-2 alone as an efficient inducer for differentiation of human bone marrow mesenchymal stem cells into dopaminergic neurons. *Journal of Biomedical Science*.

[23] Mohanty S., Bose S., Jain K. G., Bhargava B., Airan B. (2013). TGF*β*1 contributes to cardiomyogenic-like differentiation of human bone marrow mesenchymal stem cells. *International Journal of Cardiology*.

[24] Kakkar A., Nandy S. B., Gupta S., Bharagava B., Airan B., Mohanty S. (2019). Adipose tissue derived mesenchymal stem cells are better respondents to TGF*β*1 for in vitro generation of cardiomyocyte-like cells. *Molecular and Cellular Biochemistry*.

[25] Satelli A., Li S. (2011). Vimentin in cancer and its potential as a molecular target for cancer therapy. *Cellular and Molecular Life Sciences*.

[26] Dominici M., Le Blanc K., Mueller I. (2006). Minimal criteria for defining multipotent mesenchymal stromal cells. The International Society for cellular therapy position statement. *Cytotherapy*.

[27] El-Mahgoub E. R., Ahmed E., Afifi R. A.-E. A., Kamal M.-A., Mousa S. M. (2014). Mesenchymal stem cells from pediatric patients with aplastic anemia: isolation, characterization, adipogenic, and osteogenic differentiation. *Fetal and Pediatric Pathology*.

[28] Jiang S. Y., Xie X. T., Jiang H., Zhou J. J., Li F. X., Cao P. (2014). Low expression of basic fibroblastic growth factor in mesenchymal stem cells and bone marrow of children with aplastic anemia. *Pediatric Hematology and Oncology*.

[29] Michelozzi I. M., Pievani A., Pagni F. (2017). Human aplastic anaemia-derived mesenchymal stromal cells form functional haematopoietic stem cell niche in vivo. *British Journal of Haematology*.

[30] Li J., Yang S., Lu S. (2012). Differential gene expression profile associated with the abnormality of bone marrow mesenchymal stem cells in aplastic anemia. *PLoS One*.

[31] Tripathy N. K., Singh S. P., Nityanand S. (2014). Enhanced adipogenicity of bone marrow mesenchymal stem cells in aplastic anemia. *Stem Cells International*.

[32] Wang M., Yuan Q., Xie L. (2018). Mesenchymal stem cell-based immunomodulation: properties and clinical application. *Stem Cells International*.

[33] Wu L., Mo W., Zhang Y. (2017). Vascular and perivascular niches, but not the osteoblastic niche, are numerically restored following allogeneic hematopoietic stem cell transplantation in patients with aplastic anemia. *International Journal of Hematology*.

[34] Kojima S., Matsuyama T., Kodera Y. (1992). Hematopoietic growth factors released by marrow stromal cells from patients with aplastic anemia. *Blood*.

[35] Bianco P., Gehron Robey P. (2000). Marrow stromal stem cells. *Journal of Clinical Investigation*.

